# Association between Type 1 Diabetes Mellitus and Parkinson’s Disease: A Mendelian Randomization Study

**DOI:** 10.3390/jcm13020561

**Published:** 2024-01-18

**Authors:** Aaron Shengting Mai, Brendan Jen-Wei Tan, Qiao-Yang Sun, Eng-King Tan

**Affiliations:** 1 Yong Loo Lin School of Medicine, National University of Singapore, Singapore 117597, Singapore; mai.shengting.aaron@gmail.com; 2 Department of Neurology, Singapore General Hospital Campus, National Neuroscience Institute, Singapore 308433, Singapore; 3 Neuroscience and Behavioral Disorders, Duke-NUS Medical School, Singapore 169857, Singapore

**Keywords:** Mendelian randomization, Parkinson’s disease, type 1 diabetes mellitus, FinnGen

## Abstract

While much evidence suggests that type 2 diabetes mellitus increases the risk of Parkinson’s disease (PD), the relationship between type 1 diabetes mellitus (T1DM) and PD is unclear. To study their association, we performed a two-sample Mendelian randomization (MR) using the following statistical methods: inverse variance weighting (IVW), MR-Egger, weight median, and weighted mode. Independent datasets with no sample overlap were retrieved from the IEU GWAS platform. All the MR methods found a lower risk of PD in T1DM (IVW—OR 0.93, 95% CI 0.91–0.96, *p* = 3.12 × 10^−5^; MR-Egger—OR 0.93, 95% CI 0.88–0.98, *p* = 1.45 × 10^−2^; weighted median—OR 0.93, 95% CI 0.89–0.98, *p* = 2.76 × 10^−3^; and weighted mode—OR 0.94, 95% CI 0.9–0.98, *p* = 1.58 × 10^−2^). The findings were then replicated with another independent GWAS dataset on T1DM (IVW—OR 0.97, 95% CI 0.95–0.99, *p* = 3.10 × 10^−3^; MR-Egger—OR 0.96, 95% CI 0.93–0.99, *p* = 1.08 × 10^−2^; weighted median—OR 0.97, 95% CI 0.94–0.99, *p* = 1.88 × 10^−2^; weighted mode—OR 0.97, 95% CI 0.94–0.99, *p* = 1.43 × 10^−2^). Thus, our study provides evidence that T1DM may have a protective effect on PD risk, though further studies are needed to clarify the underlying mechanisms.

## 1. Introduction

Parkinson’s disease (PD), characterized by rest tremor, bradykinesia, rigidity, and postural instability, is one of the most common neurodegenerative disorders [1]. Both motor and non-motor symptoms contribute to a poor quality of life and an increased disease burden for patients and their caregivers. Several pathogenic mutations, common risk variants, and environmental and lifestyle factors have been etiologically linked to this disease [1,2]. In particular, identifying risks or protective factors can potentially reduce or protect against the condition. 

Diabetes mellitus (DM) is one of the most common metabolic diseases, and there are >500 million adults with the condition, with an estimated prevalence of >10% among adults globally [3,4,5,6]. In type 1 DM, the pancreas does not produce insulin (mostly due to the immune system destroying islet cells), whereas in the much more common type 2 DM, there is less production of insulin, and the body develops insulin resistance, especially in people with obesity [5,6]. Long-standing, poorly controlled DM can be associated with systemic complications, including microvascular and macrovascular damage to many organs, leading to a poor quality of life and increased disease burden for patients, caregivers, and society [5,6].

The previous literature has extensively investigated the relationship between type 2 diabetes mellitus (T2DM) and Parkinson’s disease (PD). Firstly, various observational studies and meta-analyses have linked T2DM and even prediabetes to an increased risk of PD. Animal models have also revealed that various factors arising from T2DM—such as neuroinflammation and long-standing hyperglycemia—can cause neuronal death, α-synuclein aggregation, and eventually PD [7,8]. Additionally, the presence of T2DM in patients with PD can also increase the likelihood of worse disease progression in both the motor and cognitive domains [9]. The medications for T2DM are another important consideration since they have been demonstrated to impact the risk of Parkinson’s disease [10] and could potentially be used to modify disease progression. 

However, much less is known about the association between type 1 diabetes mellitus (T1DM) and PD. As compared with T2DM—which occurs due to insulin resistance and shares a close link with metabolic syndrome [11]—T1DM is an entirely different disorder that is hallmarked by the autoimmune destruction of insulin-secreting pancreatic islet beta cells [12]. Whilst generally considered to be pathophysiologically and genetically distinct, T1DM and T2DM have been shown to share certain genetic features and key pathophysiologic steps, such as the apoptosis of pancreatic islet beta cells [13]. There are important limitations in the current literature linking T1DM and PD. Firstly, many observational studies have not explicitly distinguished between T1DM and T2DM when defining diabetes mellitus as a risk factor, and secondly, T2DM is far more prevalent than T1DM [14,15,16]. The evidence concerning a link between T1DM and PD remains mixed, too. While two studies found a potentially increased risk of PD in patients with T1DM [17,18], another study found a protective effect instead [19]. 

T1DM has a profound impact on the developing brain, most likely secondary to chronic hyperglycemia as well as episodes of diabetic ketoacidosis [20,21]. Prior research has demonstrated that the dysglycemia found in T1DM is injurious to the brain and its development [20]. Diabetic ketoacidosis, of note, is an especially important cause of neurological sequelae in patients with T1DM. Such sequelae, including neurocognitive dysfunction, can occur even without clinically apparent cerebral edema [20]. However, much of this research is focused on neuropsychological aspects and brain volumes through neuroimaging [20,21]. It is still unclear what impact T1DM has on the developing brain with regards to movement disorders such as PD. 

To address this gap in knowledge, we undertook a two-sample Mendelian randomization (MR) study, using two datasets on T1DM and another dataset on PD, all of which have no sample overlap. The MR approach utilizes genetic variants, such as single nucleotide polymorphisms (SNPs), that are associated with the exposure of interest (in this case, T1DM) as instrumental variables (IVs) to draw a causal link between the exposure (T1DM) and the outcome of interest (PD) [22].

## 2. Methods

### 2.1. GWAS Datasets

Our outcome variable was PD, while our exposure variable of interest was T1DM. The other exposure variables included T2DM and fasting insulin. SNPs significantly associated with T1DM were extracted as IVs from the FinnGen dataset using the IEU OpenGWAS platform (GWAS ID: finn-b-E4_DM1) [23]. This analysis comprised 5928 cases and 183,185 controls of European ancestry. The cases and controls were determined using the coded diagnoses, per the International Classification of Diseases 10th Revision (ICD-10) codes, for either hospital discharge or cause of death. 

For PD (our outcome variable), we used a meta-analyzed dataset from the International Parkinson’s Disease Genomics Consortium (IPDGC). This dataset involved 33,674 cases and 449,056 controls of European ancestry (GWAS ID: ieu-b-7) [24]. A sensitivity analysis was conducted using IVs derived from another FinnGen dataset with stricter inclusion criteria, which excluded patients who were also diagnosed with type 2 diabetes mellitus (T2DM), such as those with double diabetes. This dataset comprised 2649 cases and 183,674 controls of European ancestry (GWAS ID: finn-b-E4_DM1_STRICT) [23]. 

Next, we replicated our findings with IVs extracted from an independent dataset on T1DM, which did not involve the FinnGen participants. This analysis was conducted by Forgetta et al. and involved 9266 cases and 15,574 controls of European descent (GWAS ID: ebi-a-GCST010681) [25]. Additionally, we utilized the FinnGen dataset on T2DM, which included 32,469 cases and 183,185 controls of European ancestry (GWAS ID: finn-b-E4_DM2) [23], as well as a publicly available GWAS by Chen et al. [26] on fasting insulin levels, which included 151,013 European participants (GWAS ID: ebi-a-GCST90002238). IVs were extracted from these datasets to analyze the relationship between fasting insulin and T2DM (as exposure variables) and PD (as the outcome variable).

### 2.2. Mendelian Randomization

We performed all analyses in RStudio (version 4.3.1) and the significance level was defined as *p* < 0.05. We utilized the *TwoSampleMR* package was used to conduct all MR-related analysis, which accesses the IEU GWAS database to obtain data automatically. We analyzed summary data using the two-sample MR approach, which uses strictly GWAS summary data for both exposures (T1DM, T2DM, and fasting insulin levels) and outcome of interest (PD). Firstly, exposure IVs were obtained, which were SNPs that demonstrated a statistically significant association with the exposure of interest (Table S1). The IVs were then filtered to ensure that each IV was independent. Next, the IVs were extracted from the outcome GWAS dataset. If data for the same SNP is not available, then linkage disequilibrium proxies are found. Lastly, the effects of the exposure and outcome SNPs were harmonized to ensure that each effect corresponds to the same allele. 

The odds ratio (OR) and 95% confidence interval (95% CI) using the inverse variance-weighted (IVW) method were then calculated. Additionally, multiple other methods were employed to assess the OR and 95% CI to ensure the robustness of our findings—namely MR-Egger, weighted median, and weighted mode—which are robust to violations for different IV conditions [27]. We then tested for horizontal pleiotropy using the MR Egger method and for heterogeneity using for the Cochran’s Q test using the MR-Egger method. 

## 3. Results

Fifteen SNPs were significantly associated with T1DM in the FinnGen T1DM dataset and were extracted as IVs (Table 1). All the MR methods found a significant association between T1DM and PD (IVW—OR 0.93, 95% CI 0.91–0.96, *p* = 3.12 × 10^−5^; MR-Egger—OR 0.93, 95% CI 0.88–0.98, *p* = 1.45 × 10^−2^; weighted median—OR 0.93, 95% CI 0.89–0.98, *p* = 2.76 × 10^−3^; and weighted mode—OR 0.94, 95% CI 0.9–0.98, *p* = 1.58 × 10^−2^). Tests for horizontal pleiotropy and heterogeneity were statistically insignificant (*p* = 0.670 and *p* = 0.344, respectively). 

The sensitivity analysis with the stricter FinnGen T1DM dataset revealed similar findings (Table 2). The model included eight SNPs, and all the MR methods revealed significant associations (IVW—OR 0.94, 95% CI 0.92–0.97, *p* = 3.68 × 10^−5^; MR-Egger—OR 0.92, 95% CI 0.87–0.98, *p* = 3.10 × 10^−2^; weighted median—OR 0.94, 95% CI 0.91–0.97, *p* = 4.16 × 10^−4^; weighted mode—OR 0.94, 95% CI 0.9–0.98, *p* = 1.57 × 10^−2^). Tests for horizontal pleiotropy and heterogeneity were similarly statistically insignificant (*p* = 0.403 and *p* = 0.754, respectively).

We replicated our findings with an independent GWAS dataset on T1DM by Forgetta et al. We extracted 45 IVs (Table 3), and the association between T1DM and PD was similarly significant (IVW—OR 0.97, 95% CI 0.95–0.99, *p* = 3.10 × 10^−3^; MR-Egger—OR 0.96, 95% CI 0.93–0.99, *p* = 1.08 × 10^−2^; weighted median—OR 0.97, 95% CI 0.94–0.99, *p* = 1.88 × 10^−2^; weighted mode—OR 0.97, 95% CI 0.94–0.99, *p* = 1.43 × 10^−2^). Tests for horizontal pleiotropy and heterogeneity were statistically insignificant (*p* = 0.320 and *p* = 0.301, respectively).

We performed additional analyses by using fasting insulin and T2DM as exposure variables. All the MR methods found no significant associations for fasting insulin (IVW—OR 0.91, 95% CI 0.55–1.5, *p* = 0.713; MR-Egger—OR 1.9, 95% CI 0.37–9.7, *p* = 0.446; weighted median—OR 0.82, 95% CI 0.43–1.58, *p* = 0.560; weighted mode—OR 0.81, 95% CI 0.3–2.15, *p* = 0.674). T2DM, however, had a statistically significant association with PD when using the MR-Egger method (OR 0.87, 95% CI 0.77–0.99, *p* = 0.041). However, this finding was not observed with the other MR methods (IVW—OR 1.02, 95% CI 0.96–1.08, *p* = 0.488; weighted median—OR 0.97, 95% CI 0.88–1.07, *p* = 0.560; weighted mode—OR 0.97, 95% CI 0.88–1.08, *p* = 0.605).

In summary, various statistical methods have demonstrated a significant protective effect of T1DM against PD, and our findings were replicated with a separate and independent GWAS dataset on T1DM. There was no statistically significant horizontal pleiotropy or heterogeneity detected in our results. Lastly, the additional analyses of fasting insulin levels and T2DM did not reveal any significant effects on PD.

## 4. Discussion

We conducted an MR analysis to investigate the potential causal links between T1DM and PD. The results of our analysis revealed a protective effect of T1DM against PD. This effect contrasts with the associated increased PD risk and increased disease progression with T2DM reported in most studies [28,29,30]. This finding aligns with the inherent differences between the two diabetes types: T1DM is characterized by an autoimmune response where the immune system targets and damages insulin-producing beta cells in the pancreas [19,31]. Conversely, T2DM, while sharing some genetic and lifestyle risk factors with T1DM, primarily stems from insulin resistance and lifestyle-related factors such as obesity, without the autoimmune component [19,32]. 

The association between T1DM and PD remains unclear, and there are a limited number of studies on the topic. Whilst there have been previous attempts, the literature remains mixed, with two studies finding an increased PD risk in T1DM [17,18] and one finding a decreased risk [19]. It should be noted, however, that these studies each employed very distinct methodologies. Klimek et al. [17] estimated the risks of comorbidity using nationwide insurance claims data; Witoelar et al. [18] employed the conjunctional false discovery rate method and found four genes that are weakly associated with both PD and T1DM in the same direction; and Senkevich et al. [19] adopted the MR approach for the causal effects of T1DM on PD and found a small but significant decrease in the OR. Though we employed two distinct T1DM datasets from Senkevich et al. [19], our findings similarly indicate a protective effect of T1DM on PD risk. These findings should then be replicated using additional GWAS data from large, curated biobanks, including those of non-European ancestries, to determine if they are generalizable to all patients with T1DM regardless of sociodemographic factors.

Insulin, once thought to be a peripherally acting hormone responsible for glucose homeostasis, has been shown to be involved in cellular homeostasis in the brain, including neurotransmission and cellular survival [33]. Insulin resistance in the brains of PD patients, resulting in defective insulin signaling pathways, may be linked to PD pathogenesis [33,34]. Pharmacologic studies have shown that anti-diabetic drugs can improve PD motor symptoms, and there are various ongoing drug trials in PD and Alzheimer’s disease [34]. However, based on our observations, it is not entirely clear what exact underlying pathophysiologic mechanisms contribute to the divergent clinical association study findings in T1DM and T2DM. One study used an integrated functional genomics approach to evaluate the tissue-specific impacts of SNPs that are associated with both types of DM [13]. They found overlapping genes and pathways but did not identify any correlation between the effects conferred by T1DM and T2DM risk variants [13]. Though they reported that a high-risk genetic profile for T1DM modulates the biological pathways that predispose to both types of DM, the converse is not true for a high-risk genetic profile for T2DM, suggesting that the pathophysiologic links between the two subtypes are likely to be complex [34]. 

Interestingly, Sanz et al. [35] generated a Drosophila model of T1DM based on insulin deficiency and found that these flies displayed insulin deficiency, increased levels of carbohydrates and glycogen, and reduced activity of insulin signaling, which are features of T1DM. These flies developed motor defects, reduced tyrosine hydroxylase (seen in dopamine neurons), and increased oxidative stress in their brains, mirroring the pathophysiology of PD in humans. The authors concluded that T1DM may be a PD risk factor. In a separate study in a type 1 diabetic rat model, the investigators used [18F]FP-(+)-DTBZ, a radiotracer that targets VMAT2 (to measure β-cell mass and for PD diagnosis), and they found decreased [18F]FP-(+)-DTBZ uptake in the striatum in these rats. Increased glucose levels were correlated with VMAT2 expression in the striatum. They suggest that, based on the observations, DM is a risk factor for PD [36]. The divergent observations between clinical associations and functional studies need to be further addressed.

We would like to highlight that the variant rs6679677, which was significantly associated with T1DM, is situated between PHTF1 (Putative Homeodomain Transcription Factor 1) and RSBN1, which are implicated in systemic seropositive rheumatic diseases [37]. Although MR is a powerful statistical method used in epidemiology and genetics to assess causality between exposures, its effectiveness heavily depends on the critical assumption that the genetic variant serving as an IV remains unassociated with confounding factors. When this assumption is compromised, it can introduce bias into the results [38]. Furthermore, there is a limited availability of well-established genetic variants, and MR methods often face constraints due to relatively small case cohorts. Additionally, it is worth noting that MR methods may not adequately address potential nonlinear or threshold effects and may not fully account for the intricacies of gene–environment interactions [39]. To enhance the reliability of the findings and draw more robust conclusions regarding causal relationships, larger and more comprehensive studies to examine the link between T1DM and PD are needed.

Our study has some inherent limitations. Firstly, the datasets employed in our study all involved only patients of European ancestry, which limits its generalizability to other important demographics, such as patients of African or Asian ancestry. To address this limitation, large biobanks for Asian and other non-European populations should be developed, and the data should be linked with available R packages. Secondly, we were unable to analyze the impact of T1DM on other PD parameters, such as disease progression and symptomatology, as these datasets did not have such information. The integration of longitudinal clinical data with genomic and other multi-omic data would be helpful in studying the evolution of a disease over time, deriving biomarkers, and identifying different subgroups. Lastly, as with all studies that utilize genomic data, one limitation of the present study is the possibility of survival bias. That said, though patients with T1DM have a lower life expectancy than the general population, most patients still live to late adulthood (65 years of age or older) [40]. 

In conclusion, our MR study, using large independent GWAS datasets, demonstrated an association between T1DM and PD risk. Clinicians should therefore maintain a high index of suspicion for PD when patients with T1DM report or exhibit tremor or other signs of rigidity and bradykinesia. Further functional studies may unravel common pathophysiologic clues and further our understanding of the underlying mechanisms, potentially identifying novel biomarkers and therapeutic targets. Next, the integration of high-quality clinical data with large sample sizes, such as longitudinal data and symptomatology, would be helpful in studying the different aspects of this association. Lastly, future studies should also investigate whether the treatment and control of T1DM impact PD risk. If so, T1DM treatment could be repurposed to lower PD risk or slow PD progression in patients with T1DM.

## Figures and Tables

**Table 1:** Instrumental variables for Mendelian randomization of the effect of type 1 diabetes mellitus on Parkinson’s disease.

SNP	Beta		Standard Error		*p*	
	Exposure	Outcome	Exposure	Outcome	Exposure	Outcome
rs116039340	−0.5196	0.0074	0.0514	0.048	4.92 × 10^−24^	0.8773
rs1611236	−0.1823	0.0012	0.0258	0.0197	1.7 × 10^−12^	0.9509
rs183697542	−0.6317	−0.1616	0.0918	0.1133	5.88 × 10^−12^	0.1538
rs3129871	0.7752	−0.0345	0.023	0.0199	1 × 10^−200^	0.08267
rs3184504	−0.1646	−0.0046	0.0212	0.0167	8.97 × 10^−15^	0.7826
rs34337125	−0.1173	0.0389	0.0213	0.0236	3.51 × 10^−8^	0.09883
rs41173	−0.1354	0.019	0.0223	0.0177	1.31 × 10^−9^	0.281
rs6679677	0.4475	−0.0377	0.0295	0.0292	8.24 × 10^−52^	0.1972
rs689	0.399	−0.0581	0.0274	0.0201	5.29 × 10^−48^	0.003846
rs705699	0.1409	0.0036	0.0215	0.0178	5.24 × 10^−11^	0.8415
rs74203920	0.3587	0.0332	0.0549	0.0721	6.63 × 10^−11^	0.6453
rs76573413	−0.7019	0.0676	0.1179	0.0965	2.6 × 10^−9^	0.4836
rs9264277	0.2268	−0.0065	0.0239	0.0189	2.73 × 10^−21^	0.7311
rs9275183	0.9606	−0.1078	0.0256	0.0301	1 × 10^−200^	0.000346
rs9468618	−0.3484	−0.0087	0.0483	0.0309	5.55 × 10^−13^	0.777

**Table 2:** Instrumental variables for Mendelian randomization of type 1 diabetes mellitus (stricter definition) on Parkinson’s disease.

SNP	Beta		Standard Error		*p*	
	Exposure	Outcome	Exposure	Outcome	Exposure	Outcome
rs1233386	0.4512	0.0006	0.042	0.0325	6.74 × 10^−27^	0.9856
rs1611236	−0.2225	0.0012	0.0377	0.0197	3.77 × 10^−9^	0.9509
rs6679677	0.5854	−0.0377	0.0434	0.0292	2.03 × 10^−41^	0.1972
rs689	0.5794	−0.0581	0.041	0.0201	2.58 × 10^−45^	0.003846
rs9260231	−0.5773	0.0428	0.065	0.0321	6.65 × 10^−19^	0.1826
rs9264277	0.3033	−0.0065	0.035	0.0189	4.39 × 10^−18^	0.7311
rs9268833	1.1716	−0.0712	0.0378	0.0234	1 × 10^−200^	0.002343
rs9468618	−0.4476	−0.0087	0.0717	0.0309	4.28 × 10^−10^	0.777

**Table 3:** Instrumental variables for Mendelian randomization of type 1 diabetes mellitus (replication dataset) on Parkinson’s disease.

SNP	Beta		Standard Error		*p*	
	Exposure	Outcome	Exposure	Outcome	Exposure	Outcome
rs10183097	0.2053	−0.0077	0.0322	0.0281	1.82 × 10^−10^	0.7851
rs1027769	−0.9962	0.1736	0.1588	0.224	3.52 × 10^−10^	0.4384
rs10760335	0.1357	−0.0031	0.0243	0.0237	2.43 × 10^−8^	0.8961
rs10774624	−0.2556	−0.0022	0.0244	0.0179	1.34 × 10^−25^	0.9037
rs10774624	−0.2556	−0.0022	0.0244	0.0179	1.34 × 10^−25^	0.9037
rs10830227	0.1582	−0.0046	0.0233	0.019	1.02 × 10^−11^	0.8084
rs10865468	−0.1624	0.0157	0.0277	0.0223	4.66 × 10^−9^	0.483
rs10911399	−0.3707	0.024	0.064	0.0513	6.75 × 10^−9^	0.6407
rs1131017	−0.2461	−0.011	0.0238	0.0174	4.24 × 10^−25^	0.5274
rs1131017	−0.2461	−0.011	0.0238	0.0174	4.24 × 10^−25^	0.5274
rs11571297	−0.1964	0.0397	0.0237	0.017	1.11 × 10^−16^	0.01962
rs12722495	−0.3145	−0.0348	0.0408	0.0288	1.27 × 10^−14^	0.2268
rs13182737	0.1465	−0.0382	0.0259	0.025	1.49 × 10^−8^	0.1264
rs17125653	0.2355	0.0383	0.0402	0.0406	4.75 × 10^−9^	0.3454
rs17863786	0.4144	−0.0964	0.0628	0.0552	4.26 × 10^−11^	0.080921
rs185774696	0.6489	−0.1542	0.0418	0.0659	2.66 × 10^−54^	0.01924
rs1869449	0.1769	0.0067	0.0269	0.0244	4.55 × 10^−11^	0.7838
rs192324744	0.562	−0.0214	0.0875	0.0943	1.36 × 10^−10^	0.8205
rs194749	−0.1638	−0.0052	0.0281	0.021	5.37 × 10^−9^	0.8052
rs201417739	−0.416	0.0699	0.0663	0.1002	3.41 × 10^−10^	0.4854
rs202520	−0.1573	0.0025	0.0256	0.021	7.97 × 10^−10^	0.9068
rs206763	0.6792	0.0407	0.0779	0.0623	2.93 × 10^−18^	0.5133
rs2071647	0.1526	−0.0127	0.0258	0.0202	3.3 × 10^−9^	0.5284
rs2111485	0.1577	−0.0209	0.0248	0.0175	1.89 × 10^−10^	0.2322
rs2144013	0.2234	−0.0339	0.0317	0.0219	1.76 × 10^−12^	0.1214
rs2269247	0.1709	0.0431	0.0295	0.0223	7.28 × 10^−9^	0.052811
rs2269247	0.1709	0.0431	0.0295	0.0223	7.28 × 10^−9^	0.052811
rs231971	0.2411	0.0061	0.0399	0.0434	1.55 × 10^−9^	0.8879
rs34536443	−0.4139	−0.0547	0.0665	0.0447	4.84 × 10^−10^	0.2213
rs4566101	0.1755	0.0154	0.0255	0.0253	6.23 × 10^−12^	0.542199
rs506770	1.0048	−0.0233	0.0426	0.0251	3.3 × 10^−123^	0.3546
rs55996894	−0.1785	0.035	0.0323	0.0302	3.13 × 10^−8^	0.247
rs59680223	0.6421	0.1726	0.1032	0.153	5 × 10^−10^	0.2593
rs62410259	−0.3796	−0.058	0.0533	0.044	1.02 × 10^−12^	0.1879
rs6679677	0.6527	−0.0377	0.0346	0.0292	3.42 × 10^−79^	0.1972
rs6719660	0.2918	0.0027	0.0524	0.0454	2.52 × 10^−8^	0.9534
rs689	0.7004	−0.0581	0.0354	0.0201	2.3 × 10^−87^	0.003846
rs6909461	−0.314	0.0064	0.0332	0.0206	3.06 × 10^−21^	0.754401
rs741172	−0.2034	−0.0059	0.0258	0.0181	3.11 × 10^−15^	0.743201
rs77523242	−0.3705	−0.0394	0.0635	0.0555	5.42 × 10^−9^	0.4777
rs79075295	−0.4192	0.051	0.0621	0.102	1.46 × 10^−11^	0.616799
rs8056814	0.2641	−0.0348	0.0415	0.0305	1.99 × 10^−10^	0.2547
rs9273363	1.2786	−0.0476	0.0334	0.0219	1 × 10^−200^	0.02981
rs9296062	0.6913	0.0139	0.054	0.044	1.37 × 10^−37^	0.751299
rs9468618	−0.3009	−0.0087	0.0489	0.0309	7.53 × 10^−10^	0.777

## Data Availability

Code and extracted data are available on request. All the data analyzed can be found in already published studies, and no new original data were generated or analyzed in this study.

## Supplementary Materials

The following supporting information can be downloaded at: https://www.mdpi.com/article/10.3390/jcm13020561/s1, Table S1: Attributes of Datasets Used.
