# Supplementary material for: Association between Type 1 Diabetes Mellitus and Parkinson’s Disease: A Mendelian Randomization Study
Source: J Clin Med. 2024 Jan 18;13(2):561. doi: 10.3390/jcm13020561 (PMC10816052; doi:10.3390/jcm13020561)
Supplement: Supplementary file 1 [file jcm-13-00561-s001.zip › jcm-2770473-supplementary.pdf]

Table S1: Attributes of Datasets Used.

Dataset	GWAS ID[a]	Source	Year	Sample Size	Number of SNPs
Type-1 DM	finn-b-E4_DM1	FinnGen	2021	Cases: 5,928<br>Controls: 183,185	16,380,008
Type-1 DM (strict)	finn-b-E4_DM1_STRICT	FinnGen	2021	Cases: 2,649<br>Controls: 183,674	16,380,237
Type-1 DM (replication)	ebi-a-GCST010681	Forgetta et al	2020	Cases: 9,266<br>Controls: 15,574	12,783,129
PD	ieu-b-7	Nalls et al	2019	Cases: 33,674<br>Controls: 449,056	17,891,936
Fasting insulin	ebi-a-GCST90002238	Chen et al	2021	151,013	29,664,438
Type-2 DM	finn-b-E4_DM2	FinnGen	2021	Cases: 32,469<br>Controls: 183,185	16,380,440

Abbreviations: DM, diabetes mellitus; GWAS, genome-wide association study; PD, Parkinson's disease; SNP, single nucleotide polymorphisms.
a. This ID can be input into the web interface of the IEU OpenGWAS project (https://gwas.mrcieu.ac.uk/datasets/) to obtain the dataset used.
